# Adaptation and Validation of the Brief Sexual Opinion Survey (SOS) in a Colombian Sample and Factorial Equivalence with the Spanish Version

**DOI:** 10.1371/journal.pone.0162531

**Published:** 2016-09-14

**Authors:** Pablo Vallejo-Medina, Laurent Marchal-Bertrand, Mayra Gómez-Lugo, José Pedro Espada, Juan Carlos Sierra, Franklin Soler, Alexandra Morales

**Affiliations:** 1 SexLabKL, Fundación Universitaria Konrad Lorenz, Bogotá, Colombia; 2 School of Psychology, Universidad Miguel Hernández, Elche, Spain; 3 Mind, Brain and Behavior Research Center (CIMCYC), Universidad de Granada, Granada, Spain; 4 School of Psychology, Universidad del Rosario, Bogotá, Colombia; Universidade Federal do Rio de Janeiro, BRAZIL

## Abstract

Attitudes toward sexuality are a key variable for sexual health. It is really important for psychology and education to have adapted and validated questionnaires to evaluate these attitudes. Therefore, the objective of this research was to adapt, validate and calculate the equivalence of the Colombia Sexual Opinion Survey as compared to the same survey from Spain. To this end, a total of eight experts were consulted and 1,167 subjects from Colombia and Spain answered the Sexual Opinion Survey, the Sexual Assertiveness Scale, the Massachusetts General Hospital-Sexual Functioning Questionnaire, and the Sexuality Scale. The evaluation was conducted by online and the results show adequate qualitative and quantitative properties of the items, with adequate reliability and external validity and compliance with the strong invariance between the two countries. Consequently, the Colombia Sexual Opinion Survey is a valid and reliable scale and its scores can be compared with the ones from the Spain survey, with minimum bias.

## Introduction

In 1976, Byrne [1] included a proposal in his pattern of sexual behavior sequence, whereby a negative—affective evaluation toward sex could reduce the likelihood of any type of preparatory sexual behavior. This proposal would generate a biased viewpoint about sexuality, thus limiting sexual communication. Subsequently, this theory has been extended from erotophobia—erotophilia.

Erotophobia-erotophilia is the learned attitude to respond to sexual stimuli from an affective evaluation performed in a negative (erotophobia)—positive (erotophilia) continuum [2]. This construct is widely studied for its positive and negative direct implications for sexual health. It is directly related to three key components of sexual health: sexual risk behavior [3,4], sexual function [5–8] and sexual victimization, although the implication is not clear yet. It in fact seems that, if the victimization occurs during childhood or adolescence, women score higher in erotophilia [9,10], but if this abuse occurs in adulthood they score higher in erotophobia [11]. In addition, erotophobia—erotophilia is related to other sexual variables. Accordingly, erotophobia—erotophilia relationships with a wide range of other sexual affective-cognitive appraisal have been observed, i.e. some aspects of the self [12], sexual cognitions [13,14], seeking of sexual sensations [15,16] and sexual fantasies [6,17] among others. Finally, erotophobia—erotophilia relationship with other constructs of great importance for sexual health such as sexual arousal / inhibition [16,18], sexual assertiveness [8], body image [19] and sexual compulsions [18] have also been observed.

Erotophobia-erotophilia has been extensively evaluated with the Sexual Opinion Survey [2]. This scale consists of 21 items that evaluate affective response towards different types of sexual stimuli (heterosexual, homosexual, autoerotic behavior, sexual fantasies and sexual stimuli). The Sexual Opinion Survey is a reliable scale (Cronbach's α = .86 [2] and .82 [20]). However, this scale has not completely shown a clear dimensionality. Different authors [2, 21–27] have observed dimensionalities which vary in number of factors and contents through samples and cultures. Nonetheless, this scale has been used dimensionally in almost all cases. Vallejo-Medina, Granados, and Sierra (2014) evaluated the items qualitatively in a study conducted in Spain [28]. Apparently, 15 items from the original 21 did not to assess the erotophobia—erotophilia construct. Consequently, Vallejo-Medina and colleagues [28] as different authors [29,30] proposed a brief version of the scale which confirmed the dimensionality of the scale (using the Structural Equation Modeling) and showed adequate psychometric properties.

Given the importance of the construct, the objective of this instrumental study [31] was to adapt and validate the Brief Sexual Opinion Survey in a sample of Colombian men and women, as well as to verify its factorial equivalence between the Spanish and Colombian versions, since this construct is based on cultural standards [32].

## Method

### Participants

The sample of this study consists of three groups:

The first sample consisted of four psychologists who had at least a graduate degree. All of the psychologists were Colombian born/residents and had been living and studying in Spain for at least two years. They were responsible for the cultural adaptation of the scale from the Spanish version to the Colombian version (see procedure and/or S1 File).

The second sample consisted of four Colombians; all of them were experts in sexuality and / or psychometrics and they evaluated four properties of the items (i.e. representativeness of the construct, understanding, interpretation and clarity).

Lastly, the third sample included a total of 1,167 subjects. From this sample, 646 participants were Colombian and 521 participants were Spanish. Furthermore, 68.40% of the Colombian participants evaluated lived in Bogotá, followed by 9% who lived in Ibague, 2.8% in Medellin and 1.5% in Pereira. The other participants were distributed in 55 different cities. In Spain, 26.1% of the participants lived in Elche, 22.1% in Alicante, 6% in Madrid, 5.4% in Granada and 3.1% in Barcelona. The other participants were evaluated in 81 different cities. Distribution of the sample under socio-psycho-sexual and demographic information is shown in Table 1.

**Table 1:** Demographic and socio-psycho-sexual description of the sample.

Variables	Colombia	Spain	Hypothesis contrast
Sex			
Female	397	313	*χ*^2^ (1) = 0.15; *p* = .69
Male	242	200	* *
Age	32.08(10.86)	34.13(12.88)	*t*(1165) = 2.94; *p* < .01; *d* = 0.17
Years of schooling	16.70(2.85)	15.77(4.07)	*t*(1154) = 4.53; *p* < .01; *d* = 0.26
Sexual Orientation			
1	545	432	
2	45	38	* *
3	8	5	* *
4	9	6	*χ*^2^ (8) = 5.90; *p* = .65
5	2	4	* *
6	8	12	* *
7	20	20	* *
8	4	3	* *
Relationship			
No	187	149	*χ*^2^ (1) = 0.38; *p* = .84
Yes	454	371	* *
Marital status			
Single	361	313	
Married	147	144	* *
Common-law marriage	87	30	*χ*^2^ (3) = 23.28; *p* < .01; *η* = 0.09
Divorced	46	25	* *
Religiousness			
Daily.	7	1	
Once a week.	101	13	* *
Rarely in a month.	139	23	*χ*^2^ (5) = 178; *p* < .01; *η* = 0.37
Rarely in a year.	217	194	* *
Never.	172	287	* *

Note. *M* = Mean; *SD* = Standard Deviation; Sexual Orientation 1 = completely heterosexual, 4 = same number of contacts either heterosexuals or homosexuals, 7 = completely homosexual, 8 = asexual.

### Instruments

#### Sexual Opinion Survey (SOS; Fisher et al, 1988 [2].)

This research was based on the brief version of the SOS scale adapted for Spain [28]. The aforementioned scale has proven validity and reliability in Spain, it consists of 6 items answered on a 7-answer Likert scale. High scores mean high erotophilia. The published version of the scale was applied for the Spanish subsample, while the adaptation of the scale made in this study, was used for the Colombian subsample. See Appendix.

#### Sexual Assertiveness Scale (SAS; Morokoff et al, 1997 [33].)

A brief 9—item version of the SAS scale validated in Spain and in Colombia [34] was used in this study (from the 18—item original version only the items which were positively worded were included). The brief SAS evaluates 3 dimensions: *Initiation*, which is the ability to initiate sexual relations whenever it is wanted, as well as carry them out as desired; *refusal*, conceptualized as the ability to reject sexual unwanted practices or contact; and Sexually Transmitted Diseases—unwanted Pregnancy (STD-P), which evaluates the ability to negotiate the use of a condom. Each dimension consists of 3 items answered on a 5—alternative Likert scale ranging from 0 = *never* to 4 = *always*. The Colombian version of the SAS was adapted and validated based on the Spanish version which had been previously validated [35,36]. High scores mean higher sexual assertiveness. Adequate reliabilities for the three subscales (Initiation = .72 and .75; Refusal = .60 and .82; STD-P = .90 and .91 for Colombia and Spain respectively) were observed in this study.

#### Massachusetts General Hospital-Sexual Functioning Questionnaire (MGH-SFQ; Labbate & Lare, 2001[37])

The Spanish versions validated in Spain [38] and in Colombia [39] were used in this study. The MGH-SFQ is a questionnaire that briefly evaluates sexual functioning during the past month. The following five dimensions are evaluated in men: *Sexual interest*, *sexual arousal*, *orgasm*, *erection and general sexual satisfaction;* and four dimensions in women (the same dimensions excluding erection). All dimensions are composed of a single item and the scale can be interpreted as one-dimensional (General Sexual Functioning) or multidimensional. The questionnaire is answered using a 5—alternative Likert scale (0 = *Totally reduced* and 4 = *Normal*). An example of an item is *How was your ability to reach an orgasm during the last month*? Reliability in Colombia and Spain was .88 and .89 for women and .89 and .81 for men in this study. High scores indicate better sexual functioning.

#### Sexuality Scale (SS; Snell and Papini, 1989 [40])

The 15-item brief version of Wiedemann and Allgeier (1993) [41] was used. Participants were evaluated using the validated Spanish versions of Spain and Colombia [42]. The SS is answered in a 5 -category Likert scale ranging from Strongly disagree, to Strongly Agree. The questionnaire evaluates three different dimensions: *Sexual Self-Esteem*, *Sexual Depression* and *Sexual Preoccupation*; each dimension consists of 5 items. Reliability of the subscales is adequate; the Cronbach α ranges from 85–93 and the test-retest reliability obtained minimum significant correlations of .67. Besides, the external validity of the scale has proved to be adequate in English speaking contexts. Appropriate Cronbach alphas for Sexual Self-Esteem (82 in Colombia and .87 in Spain), Sexual Depression (.85 in Colombia and .86 in Spain) and Sexual Preoccupation (.85 in Colombia and. 85 in Spain) have been observed in this study.

### Procedure and data analysis

The cultural adaptation of the SOS to Colombia was performed based on its brief version, which was validated in Spain [28] following the protocol to adapt instruments from one language to the same language in another culture as recommended by Vallejo-Medina et al., [34]. This protocol is based on Muniz et al.’s guidelines [43], albeit the indications of APA, AERA, NCME (2014) [44] and Elosua et al. (2014) [45] were also considered. Adaptation was conducted by four Colombian psychologists, all of whom held a graduate degree and had lived in Spain, along with our team of researchers (See Vallejo-Medina et al. [3. 4] for further information).

Once the initial adapted version was ready, a total of four different psychometrics and / or sexuality experts evaluated the adaptation of the items. All the experts evaluated *Representativeness* of the item to the erotophobia-erotophilia construct, *Understanding* the item in the Colombian version, a single interpretation (no ambiguity), and Clarity of the item (how concise it is). To this end, a table of specifications of the items [46] and the ICaiken [47] program–which allows to obtain the confidence interval for the Aiken V–[48,49] were used. Experts scored the property of each item in a range of 1 (*Nothing*) to 4 (V*ery*). A cut point below .50 in the lower limit (CI = .95%) of the Aiken V has been considered as a criterion of item inadequacy [47].

The main sampling was conducted similarly in both countries. Evaluation began on October 23, 2014 and ended on February 24, 2015. Sampling was incidental and the evaluation was made online. Questionnaires were designed in Typeform and distributed through personal and Facebook main researchers contacts and the official universities Facebook profiles. There were 1,114 single pageviews to the questionnaire site in Spain, of which only 576 people answered the questionnaires completely (51.70% of those who accessed the website). 40.62% of the sample answered the questionnaires from laptops or PCs, 31.42% from tablets and 27.77% from smartphones. The average time to finish the questionnaires was 19 minutes and 12 seconds. There were 2,498 single pageviews in Colombia, of which 48.95% (1,223) answered all the questionnaires; 707 (57.80%) were answered from PCs or laptops, 56 (4.57%) from tablets and 451 (36.87%) from smartphones. The average time to answer the questionnaires was 13 minutes and 18 seconds. The initial Colombian participants were younger, so this part of the sample was reduced by half; therefore, 500 young participants were excluded (the other participants from Spain and Colombia were eliminated after noticing errors in their answers). In order to control repetitions or double cases we have checked the IP´s and did a cross validation with sex, age and data of submission.

EQS 6.1. was used to calculate Factorial Invariance (FI). This was evaluated progressively under a Mean and Covariances Structures procedure (MACS) as recommended [50]. This procedure allows a strong evaluation of invariance against Covariances Structures Analysis (COVS), which only allows a weak evaluation of the FI [51]. A Moment analysis and Maximum Likelihood, Robust (ML, Robust) were used. The latter is a robust estimator given noncompliance with the multivariate normality. (Mardia = 36.5 in Colombia and 47.4 in Spain). Progressive FI was performed in four steps: 1) *Configural invariance* (invariance will be evaluated without restrictions in the model); 2) *Metric or weak invariance* (the factorial weights will be restricted by assessing the equivalence of the weight of each item with regard to the factor); 3) *Strong invariance* (the intercepts will be restricted); and 4) *Strict invariance* (the variances of errors will be restricted). The overall fit indices used were the Root Mean Square Error Approximation (RMSEA) [52] and its 90% confidence interval, as well as the Comparative Fit Index (CFI) [53]. Values lower than .06 for the RMSEA [54] and higher than .95 for the CFI will be considered of good fit. In addition, the CFI will be the main indicator used to evaluate the FI; the fact that the CFI does not decrease by more than .01 compared to the previous model [55] shall be considered as evidence of invariance. Finally, Akaike Information Criterion (AIC) [56] will also be reported. A considerable increase in this indicator will be a sign of absence of FI.

The other results were obtained using the SPSS 20.0.

This work was reviewed and approved by an independent ethical committee (Research and Ethics Committee of Psychology School) of our institution (*Fundación Universitaria Konrad Lorenz*) in compliance with the 1975 Declaration of Helsinki, as revised in 1983 Ethic Committee for Clinical Research. It was approved within the document "*Informes de Investigaciones 2014 Acta número 2014–003*". All participants provided write informed consent. The ethical committee reviewed the consent procedure, but did not review the informed consent itself.

## Results

### Item Analysis

Table 2 shows the qualitative evaluation that four experts in psychometrics and / or sexuality performed on the 6 items from the Colombian version. The data show an appropriate wording of the items of the brief Colombian version, with a 95% lower limit of the Aiken V, which was always above .50.

**Table 2:** Qualitative properties of the items.

								V Aiken	
Item	Prop	EXP1	EXP2	EXP3	EXP4	*M*	V Aiken	Ll 95%	Ul 95%
	Rep	3	4	4	4	3.75	.92	.64	.98
Item 1	Und	3	4	4	4	3.75	.92	.64	.98
	Inter	4	4	4	4	4	1.00	.75	1.00
	Clar	3	4	4	4	3.75	.92	.64	.98
	Rep	3	4	4	4	3.75	.92	.64	.98
Item 2	Und	4	4	4	4	4	1.00	.75	1.00
	Inter	4	4	4	4	4	1.00	.75	1.00
	Clar	4	4	4	4	4	1.00	.75	1.00
	Rep	3	4	4	4	3.75	0.92	.64	.98
Item 3	Und	4	4	4	3	3.75	.92	.64	.98
	Inter	4	4	4	4	4	1.00	.75	1.00
	Clar	3	4	4	3	3.5	.83	.55	.95
	Rep	3	4	4	4	3.75	.92	.64	.98
Item 4	Und	3	4	4	4	3.75	.92	.64	.98
	Inter	4	4	4	4	4	1.00	.75	1.00
	Clar	4	3	4	4	3.75	.92	.64	.98
	Rep	3	4	4	4	3.75	.92	.64	.98
Item 5	Und	3	4	4	4	3.75	.92	.64	.98
	Inter	4	4	4	4	4	1.00	.75	1.00
	Clar	4	3	4	4	3.75	.92	.64	.98
	Rep	3	4	4	4	3.75	.92	.64	.98
Item 6	Und	3	4	4	4	3.75	.92	.64	.98
	Inter	4	4	4	4	4	1.00	.75	1.00
	Clar	4	4	4	4	4	1.00	.75	1.00

Note. Ll = Lower limit .95 confidence interval; Ul = Upper limit .95 confidence interval.

### Reliability and analysis of some psychometric properties of the items

Table 3 shows that the analyzed indicators are adequate in general. Both versions are reliable, although the α is lower in Spain. Corrected item-total correlations (*r*_it_^c^) are always above .30, except for item 1 of the Spanish version. Finally, a substantial increase of Cronbach's alpha is not observed if any item is removed, and the mean values of the items are above the theoretical mean value of the answers (4) as would be expected in non-clinical population. Furthermore, SD’s between 1 and 2 are indicative of adequate variability of the answers.

**Table 3:** Psychometric properties of the items.

Country	Item	*M*	*SD*	*r*_it_^c^	α-item	α	Total *Sum*	*M (SD)*
Colombia	SOS1	6.33	1.33	.34	.87			
	SOS2	5.28	2.02	.74	.80			
	SOS3	5.81	1.55	.68	.82			
	SOS4	4.95	2.06	.77	.80	.85	32.50	5.41 (1.80)
	SOS5	5.09	1.96	.69	.81			
	SOS6	5.03	1.93	.61	.83			
Spain	SOS1	5.88	1.72	.29	.67			
	SOS2	6.35	1.19	.50	.61			
	SOS3	6.06	1.45	.56	.58	.67	35.91	5,98 (1.53)
	SOS4	5.99	1.46	.52	.59			
	SOS5	6.00	1.65	.31	.67			
	SOS6	5.62	1.75	.33	.66			

*Note*: *Total Sum = Total Sum; M* = Mean; *SD* = Standard Deviation

r_it_^c^ = corrected total-item correlations; α-item = Cronbach alpha if item is deleted: α = Cronbach alpha.

### Factorial invariance

Progressive factorial invariance was subsequently tested, in order to give evidence of construct validity of the scale and test the possibility to compare the results between Spain and Colombia. The fitting of the four models tested can be seen in Table 4. The configural—without constrictions–model, regarded as the most basic indicator of invariance, was the first tested. A poor fit would indicate that the forms are not equivalent and would stop the sequence of progressive analysis. This first level of invariance was tested in a nested one-dimensional model for Spain and Colombia, with covariances between the errors of items 2 and 4 (E2, E4)—as both allude to autoerotic behavior of masturbation- and with covariances of errors in items 5 and 6 in Spain (E5, E6) as these are the only items worded backwardly. The first model tested indicated an excellent fit of the data matrix upon the proposed theoretical model (see Table 4). Subsequently, weak invariance was tested with constraints on λ, i.e. the factor loading equality between Spain and Colombia was proved. As shown, the level of invariance is met, with a CFI exactly equal to the one previously observed. Besides, the general adjustment in this model is again excellent. Afterwards, intercepts equality (indicator intercept equality) was proved and a strong level of invariance was found. The overall fit of the model is worse if compared to the previous model, but the reduction of the CFI remains within an acceptable range with a reduction of about .01. Moreover, the AIC increases slightly despite the significant differences between the theoretical model and the study’s data (something to be expected with these sample sizes). RMSEA–and its 90% confidence interval–and the CFI show an appropriate fit of the data. Therefore, a level of strong invariance was achieved because the variances of the errors are different, as can be noted. A level of strict invariance was not fulfilled and the progressive procedure of evaluations of the IF was stopped.

**Table 4:** Fit indexes for the progressive invariance models.

Level of invariance	S-B χ^2^	df	*p*	AIC	RMSEA	IC (90%) RMSEA	CFI	ΔCFI
Configural invariance	12.49	11	.32	-9.50	.016	.000 - .049	1.00	-
Weak invariance	18.74	17	.34	-15.26	.014	.000 - .042	1.00	.00
Strong invariance	73.70	23	.00	27.70	.063	.047 - .079	.987	- .013
Strict invariance	216.33	29	.00	158.33	.108	.095 - .122	.938	- .049

S-B χ^2^ = Satorra Bentler chi square; df = degree of freedom; AIC = Akaike Information Criterion; RMSEA = Root Mean Square Error Approximation; RMSEA IC (90%) = Confidence Interval of the RMSEA at .90; CFI Comparative Fit Index; ΔCFI = Increase of the CFI.

Standardized results of the configural model associated with factor loadings (λ), errors of the items and the variance of the item, which is explained by the single factor (erotophobia-erotophilia) can be seen in Table 5. The difference between the two backward adapted items in Spain and their equivalents in Colombia, which were not backwardly adapted, is highlighted, although apparently such difference is not so great as to prevent their comparison.

**Table 5:** Standardized factor loadings (λ), errors, and % of explained variance for the Spain and Colombia configural model.

	Colombia			Spain		
Items	λ	Error	R^2^	λ	Error	R^2^
SOS1	.36	.93	.13	.41	.91	.16
SOS2	.72	.68	.52	.54	.84	.29
SOS3	.74	.66	.55	.83	.54	.70
SOS4	.77	.62	.60	.60	.80	.36
SOS5	.79	.60	.63	.25	.96	.06
SOS6	.70	.70	.50	.34	.94	.11

Note: λ = Standardized factor loadings

R^2^ = % of explained variance.

### External validity

Table 6 shows the correlations between external criteria and the SOS. Several low significant correlations, between SOS and other criterion variables have been observed (except with Initial assertiveness, which are moderate). These correlations are similar between the two countries and always aim at the expected direction.

**Table 6:** Pearson correlation Matrix between the SOS and the criterion variables.

	Spa→	Sex Self	Sex Dep	Sex Preo	As In	As Re	As STD-P	Sex Func	SOS
↓Col									
Sex Self		1	-.55**	.04	.34**	-.01	-.06	.28**	.22**
Sex Dep		-.49**	1	.17**	-.38**	-.07	.01	-.39**	-.18**
Sex Preo		.05	.09*	1	-.02	-.21**	-.06	.19**	.10*
As In		.26**	-.26**	.09*	1	.23**	.09*	.13**	.34**
As Re		-.03	.07	-.14**	.11**	1	.19**	-.23**	.11**
As STD-P		-.12**	.06	-.09*	0.02	.22**	1	-.04	.15**
Sex Func		.31**	-.43**	.21**	.20**	-.24**	-.00	1	.17**
SOS		.12**	**.03**	.25**	.32**	**.00**	**-.03**	.08*	1

Col = Colombia; Spa = Spain; Sex Self = Sexual Self-esteem; Sex Dep = Sexual Depression; Sex Preo = Sexual Preoccupation; As In = Initiation Sexual Assertiveness; As Re = Refusal Sexual assertiveness; As STD-P = Sexual Transmitted Diseases-Pregnancy Sexual Assertiveness; Sex Func = Sexual Functioning; SOS = Sexual Opinion Survey (erotophilia-erotophobia)

* = *p* < .05

** = *p* < .01. SOS correlations are marked in bold.

### Cultural differences and percentile ranking scores

Results have shown differences which are statistically significant *t* (1127) = 7.83; *p* < .01; *d* = 0.47, between Spain (*M* = 35.91; *SD* = 5.75) and Colombia (*M* = 32.49; *SD* = 8.36). By contrast, no significant differences by sex were observed either in Spain *t* (501) = .25; *p* = .50, men (*M* = 36.04; *SD* = 5.51) and women (*M* = 35.90; *SD* = 5.84) or in Colombia *t* (609) = .40; *p* = .68 men (*M* = 32.61; *SD* = 8.08) and women (*M* = 32.33; *SD* = 8.57). Lastly, statistically significant differences in age ranges with a moderate effect size were observed in Spain *F* (2, 508) = 15.39; *p* < .01; ω = .05) as well as in Colombia *F* (2, 615) = 10.64; *p* < .01; ω = .03. Percentile Ranking Scores in three different age ranges, without sex differentiation (Table 7) were calculated for Colombia based on the aforementioned results, and considering the observed normality of data (see skewness and kurtosis).

**Table 7:** Percentile Ranking Scores for Colombia.

Country	Colombia		
Age	18–30	31–44	+45
N	296	227	95
*M*	31.32	34.50	31,34
*SD*	8.29	7.49	9,62
Skewness	-0.55	-1.25	-0.72
Kurtosis	-0.59	1.36	-0.49
Minimum	10	6	6
Maximum	42	42	42
Percentiles			
1	11	11	6
5	16	16	12
15	22	27	18
25	25	31	24
35	29	33	30
50	33	36	33
65	36	39	37
75	38	41	40
85	40	42	42
95	42	42	42
99	42	42	-

N = Sample size; *M* = Mean; *SD* = Standard Deviation

## Discussion

A brief version of the SOS was validated in Colombia in this study, and its factorial invariance with the Spanish version was proved. The results showed adequate quantitative and qualitative properties with proper reliability—though a little low for Spain. Invariance between the Spanish version and the Colombian version was strong and its external validity was confirmed. Finally, the presentation of the percentile ranking scores make the brief SOS an optimal choice to evaluate attitudes toward sexuality in Colombia.

The sample evaluated in this study covers a large area of Colombia and Spain. In addition, the sociodemographic characteristics of the sample accurately reflect both cultures. At least, this can be seen not having found any significant differences between the two countries regarding sex, sexual orientation or relationships. Additionally, differences in variables such as religiousness and marital status were found, in a more traditional way regarding Colombia. However, statistically significant differences in age and schooling level were observed between the two countries, although the effect sizes of the differences are low; for this reason, a low impact of these variables on the results could be expected.

Results of the subjective evaluation of the items showed an optimal quality regarding the items wording and realization. All of the items scored over .50 to 95% lower limit of the Aiken V as recommended [47]. Moreover, item 1 was modified adjusting the original content from *“swimming in the nude with a member of the opposite sex would be an exciting experience”* to *“swimming in the nude with a member which I feel attracted to would be an exciting experience”* in order to remove a homophobic bias as recommended in the Spanish adaptation [34]. This modification was made only for Colombia, and it has been accepted by the expert judges–as well as the other items. Another modification between the two cultures was the direct wording of items 5 and 6 in Colombia, which were kept in their backward adaptation in Spain. We would like to call attention upon the fact that backward adaptation of the items sought to do away with acquiescence in long scales, but they lose their usefulness in brief scales such as the current one.

A quantitative evaluation of the items reinforces the idea of their quality. The mean values of the item scores are slightly higher than the theoretical mean value of the answers scale; they are always higher than 1 according to the SD’s. Likewise, distribution of responses is adequate [57], as expected in community samples. Reliability of the scale is suitable for research purposes both in Spain and in Colombia [58]. However, reliability of the Spanish scale would not be enough to use the test with a clinical-diagnostic purpose. Corrected item-total correlations (above .30, except for item 1 of the Spanish version) could indicate the suitability of the modification of item 1 performed in Colombia. Items 5 and 6 of Colombian adaptation (worded in forward direction) show better indices than the same items in the Spanish adaptation, which again highlights the right decision of modifying the wording of these items. Finally, if an item is removed, reliability of the scale is not substantially improved in any case.

Since the items appear to work well for this scale, as they always have, we subsequently tested not just the one-dimensionality of the reduced scale, but also the factorial invariance between the Colombian version and the Spanish version. According to the results, a level of strong invariance for one-dimensional model was accomplished. This would imply that not only can the items be compared individually between the two countries, but the total of the SOS can also be compared between the two countries with minimum measurement bias [59]. Besides, standardized weights show that all items are significantly related with erotophobia-erotophilia, but the backward adapted items of the Spanish version (SOS 5 and 6) are those less related as observed in corrected total-item correlations.

This study also tested the external validity of the scale evaluating cross validity. Thus, the same trend was observed in Spain and in Colombia in almost all correlations; most relations were low, significant and aimed at the expected direction. Only in Sexual Depression, Refusal and STD-P Sexual Assertiveness were significant relations observed in Spain, but not so in Colombia. Further research in Colombia would be necessary in order to understand these differences. Sexual Assertiveness in Refusal is related to sexual victimization [60], and relations between erotophobia-erotophilia with sexual victimization are complex, as mentioned before. Hence, even if the objective of this study is not to examine these relations, it would be possible to think that there is more sexual victimization during childhood in Colombia than in Spain (there are no studies to prove it to the extent known) and that this fact could neutralize the relation, if victimization in childhood is related to erotophilia and victimization in adulthood with erotophobia [9–11] as stated above. Regarding the negotiation of the use of barrier methods of contraception, no relation has been found in Colombia. In this case, the numbers of teenage pregnancy are higher in Colombia than in Spain, where 1 in every 5 women (20%) between 15 and 19 years of age are mothers (Colombian Institute of Family Welfare—*Instituto Colombiano de Bienestar Familiar*) [61] against 7% for this same group in Spain [62]. Likewise, the rate of AIDS in Colombia is higher (0.5%) than in Spain (0.4%) [63]. It would be conceivable that there are more important realities than those of erotophobia-erotophilia to explain the use of latex barriers in Colombia, although again, the data is scarce and more information is required.

Results show that Colombia has lower scores on erotophilia than Spain, which would indicate more negative attitudes toward sexuality. This study is the first cultural comparison conducted with the erotophobia and erotophilia, and it is also the first study in Colombia, to the extent known. For this reason, prudence would need to be observed; nevertheless, cultural factors may modulate this variable and its interpretations are complex due to the limited information available regarding these variables, as stated above [31]. Moreover, the implications of erotophobia-erotophilia in a developing country may differ from the related implications in developed countries. Further research should be conducted now that there is a valid and reliable scale to evaluate erotophilia-erotofobia in the Colombian population. To do so, the presentation of the percentile ranking scores should guide the interpretation of scores on a scale that has proved to be reliable and valid for the Colombian, as well as for the Spanish, population.

## Appendix

### Colombian Validated version of the *Sexual Opinion Survey-6 (SOS; Fisher*, *White*, *Byrne y Kelley*, *1988)/English translation*

1 = Totalmente en desacuerdo y 7 = Totalmente de acuerdo/ 1 = I strongly agree and 7 = I strongly disagree.

1. *Bañarme desnudo/a con una persona del sexo que me atrae podría ser una experiencia excitante*./Swimming in the nude with a member of the sex I feel attracted to would be an exciting experience.
2. *Masturbarme podría ser una experiencia excitante*./ Masturbation could be an exciting experience
3. *Me resulta excitante pensar en tener una relación sexual*./ I personally find that thinking about engaging in sexual intercourse is arousing.
4. *Sería una experiencia excitante acariciar mis genitales*./ Manipulating my genitals would be an arousing experience.
5. *Me agrada tener sueños sexuales*./ I enjoy having sexual dreams.
6. *Siento curiosidad por el material de contenido sexual (libros*, *películas)*./ I am curious about material with sexual content (books, movies).

## Supporting Information

S1 File Translation, Adaptation and Validation of the brief Sexuality Scale in Colombian and Spanish Populations. Paper under revision were the procedure is described
(DOCX)
