# Supplementary material for: Adaptation and Validation of the Brief Sexual Opinion Survey (SOS) in a Colombian Sample and Factorial Equivalence with the Spanish Version
Source: PLoS One. 2016 Sep 14;11(9):e0162531. doi: 10.1371/journal.pone.0162531 (PMC5023103; doi:10.1371/journal.pone.0162531)
Supplement: S1 File — Paper under revision were the procedure is described (DOCX) [file pone.0162531.s001.docx]

Translation, Adaptation and Validation of the brief Sexuality Scale in Colombian and Spanish Populations.

Franklin Soler^1^

Mayra Gómez-Lugo^2^, José P. Espada^3^, Alexandra Morales^3^, Juan Carlos Sierra^4^, Laurent Marchal-Bertrand^2^, Pablo Vallejo-Medina^2^.

^1^School of Medicine and Health Sciences, School of Psychology, Universidad del Rosario, Bogotá, Colombia.

^2^SexLabKL, School of Psychology, Fundación Universitaria Konrad Lorenz; Bogotá, Colombia.

^3^Department of Health Psychology, Miguel Hernández University; Elche, España

^4^ Mind, Brain and Behavior Research Center (CIMCYC), Universidad de Granada, Granada, España

[franklin.soler@urosario.edu.co](mailto:franklin.soler@urosario.edu.co)

[mayraa.gomezl@konradlorenz.edu.co](mailto:mayraa.gomezl@konradlorenz.edu.co)

[jpespada@umh.es](mailto:jpespada@umh.es)

[alexandra.moraless@umh.es](mailto:alexandra.moraless@umh.es)

jcsierra@ugr.es

[laurent.marchalb@konradlorenz.edu.co](mailto:laurent.marchalb@konradlorenz.edu.co)

[pablo.vallejom@konradlorenz.edu.co](mailto:pablo.vallejom@konradlorenz.edu.co)

**Abstract**

The Sexuality Scale is an instrument which evaluates what people think and how they feel about their own sexuality through three components: Sexual Self-Esteem, Sexual Depression and Sexual Preoccupation. Given the clinical implications and impact on sexual health after having demonstrated its relationship with various components of sexuality, and the little research on the last two components of the scale, this study undertook to translate, adapt and validate the brief version of the Sexuality Scale in a sample of 1,167 Spanish and Colombian men and women. Two versions of the instrument were obtained – one for each country. In both versions, the results indicate high levels of reliability and adequate psychometric properties of the items. Configural invariance confirmed the three-dimensional structure of the scale for the two versions. The correlations with scales which evaluate various aspects of sexuality confirm adequate levels of concurrent validity. The scales may have important implications for evaluation and intervention of various dimensions of sexual behavior.

*Keywords*: Sexuality Scale, Sexual Self-Esteem, Sexual Depression, Sexual Preoccupation, Colombia, Spanish, Human Sexuality.

Translation, Adaptation and Validation of the brief Sexuality Scale in Colombian and Spanish Populations.

Snell and Papini (1989) developed the Sexuality Scale (SS) to measure what people think and how they feel about their own sexuality through three components: Sexual Self-Esteem (SSE) (dispositional tendency to positively evaluate one’s ability to relate sexually with others), Sexual Depression (SD) (chronic tendency to feel sad and discouraged about the sexual aspects of one’s life) and Sexual Preoccupation (SP); the continuing tendency to be absorbed and obsessed with sexual thoughts and behaviors that practically prevent one from thinking about other matters (Snell, Fisher, & Schuh, 2001; Snell & Papini, 1989). SSE, SD and SP would be interrelated. For example, SSE and SD would be opposite constructs that are part of the same psychological dimension (Snell & Papini, 1989); thus, high levels of sexual self-esteem indicate low levels of sexual depression (Snell et al., 2001; Snell & Papini, 1989; Wiedemann & Allgeier, 1993). Positive relationships have been demonstrated in men between SD and SP, and the two have been related to overall self-esteem and clinical depression (Wiedemann & Allgeier, 1993). SSE has been the most widely studied construct of the three, and no evidence has been found on the study of SD and SP as isolated components of sexuality.

SSE is a key component of sexual health (Heinrichs, MacKnee, Auton-Cuff, & Domene, 2009; Robinson, Bockting, Rosser, Miner, & Coleman, 2002). This component is learned from the interactions with the family context, peer relationships and sexual history (Gaynor & Underwood, 1995). Thus, various studies have demonstrated its clinical implications and its impact on sexual health. Similarly, SSE it has been negatively associated with sexual risk behaviors (Riggio, Galaz, Garcia, & Matthies, 2014; Seal, Minichiello, & Omodei, 1997), positively associated with sexual assertiveness (Ménard & Offman, 2009; Santos Iglesias & Sierra, 2010) and negatively with sexual abuse and sexual victimization (James, 2011; Lemieux & Byers, 2008; Struckman-Johnson & Struckman-Johnson, 1994). Positive relationships have also been demonstrated between SSE and sexual functioning (Dove & Wiederman, 2000; James, 2011; Wentland, Herold, Desmarais, & Milhausen, 2009), sexual satisfaction (Carrobles, Gámez-Guadix, & Almond, 2011; Sanchez-Fuentes, Santos-Iglesias, & Sierra, 2014), with the perception of physical attractiveness (Wiederman & Hurst, 2010), body image (Blodgett & Benson, 2013; La Rocque & Cioe, 2011; Van Den Brink, Smeets, Hessen, Talens, & Woertman, 2013), sexual identity and wellbeing (Muise, Preyde, Maitland, & Milhausen, 2010) and ideals of physical appearance (Calogero & Thompson, 2009a). In addition, lack of sexual self-esteem has been observed in women who use drugs (James, 2011), women who have suffered sexual abuse in childhood (Lemieux & Byers, 2008; Van Bruggen, Runtz, & Kadlec, 2006), women suffering from cancer (Andersen, 1999; Cleary, Hegarty, & McCarthy, 2011), women with eating disorders (Calogero & Thompson, 2009b), as well as who are dissatisfied with their genital appearance (Schick, Calabrese, Rima, & Zucker, 2010) or who have had a previous negative experience (Mayers, Heller & Heller, 2003) and both men and women with physical disabilities (McCabe & Taleporos, 2003).

Scales traditionally used to evaluate the SSE have been developed in the English-speaking context, and they have limitations. For example, Finkelhor's Sexual Self-Esteem Scale (Finkelhor, 1981) and Reed's Romantic and Sexual Self-Esteem Scale (Reed, 1988) are scarcely used at present and, in the case of the first, low reliability levels have been found. The Sexual Self-Esteem Inventory (SS-EI; Zeanah & Schwarz, 1996) only assesses sexual self-esteem in women. The Sexual Self-Esteem Scale, Form B (SSES-B; Gaynor & Underwood, 1995) is complete, yet it has not been evaluated in terms of its subscales’ content or concurrent validity. Similarly, Rosenthal’s Sexual Self-Esteem Scale (RSES; Rosenthal, Moore, & Flynn, 1991) has several items which evaluate sexual self-esteem, but its psychometric properties are doubtful.

Currently, the Sexuality Scale (SS; Snell & Papini, 1989) is probably the sexual self-esteem scale which has been most widely researched at the psychometric level. The Sexuality Scale starts with a thorough definition – both syntactically and semantically – of the constructs to be evaluated; it has also shown good reliability levels, with Cronbach alphas between .91 and .92 for SSE, between .85 and .93 for SD, and between .87 and .91 for SP (Snell et al., 2001). Furthermore, the Sexuality Scale has also proved to have suitable external validity indicators. These data were obtained from studies with college students. Moreover, the SS has a factor structure that has been explored, but is yet to be confirmed (Wiedemann & Allgeier, 1993).

Research on sexual self-esteem dates back to as recently as two decades ago, and future psychological evaluations for clinical applications will involve the need to evaluate sexual self – esteem with, adapted, reliable and valid instruments. To date, no scale has been validated which evaluates sexual self-esteem in the Spanish language, neither in Colombia nor in Spain. This instrumental study (Montero & León, 2007) sought to translate, adapt and validate Snell and Papini’s (1989) Sexuality Scale in its brief version by Wiedemann and Allgeier (1993), which is composed of three 5-item sub-scales (SSE, SD and SP), in a sample of Spanish and Colombian men and women.

**Method**

**Participants**

The samples of this study are grouped into two types: a sample of experts and a sample of participants. The first sample was formed with experts in sexuality and / or psychometry (five for Colombia and four for Spain). These experts evaluated the translation – see procedure – and adaptation of the instrument translated into Spanish.

The sample of participants consisted of 1,167 people (646 from Colombia and 521 from Spain) who correctly completed the survey. Table 1 presents the socio-psycho-sexual characteristics of the participants of the two cultures. Inclusion criteria for participants were as follows: being of age, having Colombian or Spanish nationality, and being resident of one of these two countries. Participants who completed the survey and did not provide their consent were excluded, as were participants aged under 18.

**Instruments**

**Background information**. The following sociodemographic information was collected: sex, sexual orientation, whether the respondent was in a relationship, marital status, income, schooling level, religion, level of religiousness, number of sexual partners and nationality.

**Sexuality Scale (SS; Snell & Papini 1989)**. We used Wiedemann and Allgeier’s (1993) abbreviated scale, which includes 15 items answered on a 5-category Likert scale ranging from *I totally disagree* to *I strongly agree*. More information on this scale can be found in the introduction. See the scale in Appendix 1.

**The Rosenberg Self-Esteem Scale (RSES; Rosenberg, 1965, 1989)**. The Spanish version (Martin-Albo, Nuñez Navarro, and Grijalvo, 2007) and Colombian (Gomez-Lugo et al., 2015) were used. The RSES is a 10-item monofactorial scale that assess self-esteem with Likert responses ranging from 1 (*I strongly disagree*) to 4 (*I strongly agree*). The scale has confirmed its dimensional structure through a Confirmatory Factor Analysis (CFA). The RSES has proved reliable with α = .86 and obtained acceptable external validity labels with significant correlations with related scales. Higher scores in this research are indicative of better overall self-esteem.

**The Sexual Opinion Survey (SOS; Fisher, White, Byrne, & Kelley, 1988)**. The brief version validated in Spain (Vallejo-Medina, Granados, & Sierra, 2014) was used for the Spanish sample, whilst the brief version adapted and validated for Colombia (Vallejo-Medina et al., 2015) was utilized for the Colombian sample. The SOS is composed of six items to be answered in a Likert scale of seven alternatives. The scale assesses erotophobia-erotophilia (positive or negative attitudes toward sexuality) in a dimensional manner. Furthermore, the SOS has proved reliable in Spain with α = .74 and α = .85 in Colombia, while proving adequate external validity. Higher scores would be indicative of better attitudes toward sexuality.

**Sexual Assertiveness Scale** **(SAS; Morokoff et al., 1997).** The brief version validated in Spain and Colombia (Vallejo-Medina et al., 2015) was used, and it consists of nine items which evaluate three components: *Initiation* – understood as the ability to initiate sex when and as desired, *Refusal* – defined as the ability to reject unwanted sexual practices or contact, and *Sexually Transmitted Diseases - Unwanted Pregnancy* (STD-P) which evaluates the ability to negotiate condom use. Each dimension is evaluated by three items which are answered in a 5-category Likert scale ranging from 0 (*Never*) to 4 (*Always*). The SAS has been previously validated in Spain (Sierra, Santos-Iglesias, & Vallejo-Medina, 2012; Sierra, Vallejo-Medina, & Santos-Iglesias, 2011; Vallejo-Medina, & Sierra, 2015). The scale shows adequate reliability indices (*Initiation* = .72 and .75; *Refusal =* .60 and .82; STD-P = .90 and .91, respectively, for Colombia and Spain). High scores are indicative of higher sexual assertiveness.

**Massachusetts General Hospital-Sexual Functioning Questionnaire** **(MGH-SFQ; Labbate & Lare, 2001)**. The Spanish versions validated in Spain (Sierra, Vallejo-Medina, Santos-Iglesias, & Lameiras, 2012) and in Colombia (Marchal-Bertrand et al., 2015) have been used in this study. This questionnaire briefly evaluates sexual functioning of males during the past month in five dimensions (*Sexual Interest, Sexual Arousal, Orgasm, Erection and Overall Sexual Satisfaction*), and four dimensions for women (same as for men, though excluding erection). All dimensions are composed of a single item, and the scale may be interpreted as being one-dimensional (*general sexual functioning*) or multidimensional. The scale is answered using a 5-choice Likert scale (0 = *Strongly decreased* and 4 = *Normal*). Reliability in this study for women in Colombia and Spain was .88 and .89, and .89 and .81 for males, respectively. Higher scores indicate better sexual functioning.

**Procedure**

Two translators residing in each country translated the instrument from English into the Spanish language of each culture. Subsequently, the research team performed cultural adaptation of the guidelines by Muñiz, Elousa and Hambleton (2013), as well as those of AERA, APA and NCME (2014) including some recommendations by Elosua, Mujika, Almeida, and Hermosilla (2014). Two adapted scales were finally obtained; one for Colombia and one for Spain.

The translated and adapted version of each scale was evaluated qualitatively by four experts in psychometrics and / or sexuality in each country. The criteria evaluated were *Representativeness* and *Ownership* (item’s contribution to the three constructs that make up the scale); *Understanding* of the item in each country’s version, *Interpretation* (no ambiguity) and item *Clarity* (how concise it is). Experts scored the property of each item in a Likert scale ranging from 1 (*Nothing ...)* to 4 (*very ...*). The level of agreement on the criteria of Representativeness, Ownership, Understanding, Interpretation and Clarity was found through a table of specifications of the items (Osterlind, 1989) on the ICaiken program (Merino Soto & Livia Segovia, 2009), which allows to obtain the confidence interval to Aiken´s V(Aiken, 1980, 1985).

The final form of the scale was administered to all participants virtually. To this end, sampling of participants was incidental in the two countries. Data collection was carried out between October 2014 and February 2015. The battery was implemented through the application Typeform © and distributed via email and Facebook ©.

**Analysis of data**

Software programs SPSS 20.0 and EQS 6.1 were used in order to find the psychometric properties of the scale.

It was determined that a score above 50 at the lower limit (at an IC = .95%) of Aiken’s V was an indicator of the criterion or Ownership (Merino Soto & Livia Segovia, 2009).

Reliability of each subscale was calculated and validity was analyzed. Mardia’s test was used for the calculation of the multivariate distribution, whereby scores above 5 are indicative of non-normality of the data. Progressive factorial invariance (FI) was calculated in order to determine whether the scales were equivalent and comparable between the Colombian and Spanish cultures. Thus, (unrestricted) configural invariance and metric or weak invariance (restricting the factor loadings) were evaluated. To this end, Maximum Likelihood, Robust (ML, Robust; Yuan & Bentler, 2000) was used as a method of estimation. The Root Mean Square Error Aproximation (RMSEA; Hu & Bentler, 1999), its respective confidence interval at 90%, and the Comparative Fit Index (CFI; Bentler, 1990) were utilized in order to evaluate the fit of the models. The criteria for determining the existence of invariance was the fact that the IFC should not diminish more than .01 as compared to the previous model (Cheung & Rensvold, 2002).

**Ethical considerations**

The project was implemented guaranteeing the preservation of international, national and institutional regulations in relation to the protection and wellbeing of human subjects partaking in it. In this regard, and in pursuance of the Declaration of Helsinki (1964), the Belmont Report (1978), Resolution 8430 / 1993 and Act 1090 / 2006, the rights of participants and the principles of autonomy and respect to persons, beneficence, non-maleficence and justice were observed. To this end, informed consents were requested and kept, wherein all the information related to the study was disseminated taking into account the principles above and respecting voluntariness.

**Results**

**Content validity and item properties.**

Scores derived from the expert’s evaluations to the items of the two versions of the scale indicated a high level of agreement in relation to ownership for eleven of the fifteen items.

The scores of the five criteria reflected the need to make adjustments in the wording of some items of the Colombian scale in order to improve their understanding and representativeness. Wording was adjusted in 10 items in the Colombian version and three items in the Spanish version.

**Reliability and some psychometric properties of the items**

Table 2 shows reliability and some psychometric properties of the items. A Cronbach α above .83 is observed in each subscale for both versions of the instrument. The corrected total correlations (*r*_it_^c^) are above .30 in all the items, and the two versions above. No significant increase is observed in Cronbach α in any of the scales if any of the items were to be removed. The mean values of the scales are adjusted to the expected values, and deviations are indicative of adequate data variability.

**Construct validity**

The data show a multivariate non-normality as per Mardia’s index (59.58 for the Colombian version and 49.64 for the Spanish version).

FI was initiated with configural – unrestricted – invariance, and a suitable fit of the data to the theoretical model was found (RMSEA = .030; IC 90% between .022 and .037; S-B χ^2^ = 256.67, df = 166, *p* =. 00 and CFI = .986). Given the adequate fit, metric invariance was subsequently addressed restricting factor loading. A clear bias was observed in the model fit (RMSEA = .079, IC90% between .073 and .085, χ^2^ = 768.81, df = 166, *p* = .00, and CFI = .929); for this reason no progress was made onto progressive evaluation.

Table 3 shows the standardized estimates of configural model are observed, noting that the factor loadings of all items are above .30.

**External validity**

Table 4 shows the correlations amongst the three subscales, and between each of them and the test instruments, in order to obtain external validity indicators. As shown in the table below, low and moderate significant correlations have been observed between the different subscales. Furthermore, the tendency observed is similar for Colombia and Spain.

**Discussion**

This study sought to translate, adapt and validate the Sexuality Scale by Snell and Papini (1989) in its brief version by Wiedemann and Allgeier (1993), into Spanish of Colombia and Spain. After the process of translating and adapting the scale, adequate psychometric properties were observed, with good evidence of validity and reliability.

The cultural gap between Colombia and Spain explains the differences of the samples in relation to religion and marital status. Higher levels of religiousness, as well as more people belonging to Christianity and Catholicism in Colombia than in Spain, as well as more frequent involvement in common-law marriage relationships; nevertheless, no statistically significant differences were observed between the two countries in terms of sex, sexual orientation and being involved in a relationship. However, statistically significant differences were observed between the two countries in terms of years of schooling and age ranges, albeit said differences are small in terms of effect sizes.

The qualitative evaluation conducted by experts of the two countries required an adjustment of some items so as to enhance their representativeness, understanding, interpretation and clarity, in light of the specification table proposed by Osterlind (1989). Along these lines of thought, linguistic corrections were adapted to the culture of the two countries in a consistent manner with the syntactic and semantic definitions of the constructs evaluated; this contributed to the improvement of content validity in the scales (Muñiz et al., 2013).

High levels of reliability were observed in the two versions of the scales with a Cronbach α between .83 and .87, i.e. in the same line of Snell et al. (2001) or Snell and Papini (1989), thereby indicating high internal consistency. These rates exceed those of other scales such as Finkelhor's Sexual Self-Esteem Scale (Finkelhor, 1981) with α = .54; the Sexual Self-Esteem Scale, Form B (Gaynor & Underwood, 1995), in which the subscales show ranging alphas the least of which is .70; or Rosenthal’s Sexual Self-Esteem Scale (SSES; Rosenthal, 1991) with α = .69 in the *Relationship to Others* scale. The Sexuality Scale’s internal consistency coefficients make it a reliable instrument for measuring sexual self-esteem, and it can be used with both clinical and research purposes (Giordano & Rush, 2010; Mayers, Heller & Heller, 2003; Nunnally & Bernstein, 1995). All items have shown adequate psychometric properties. Both the version for Spain and the version for Colombia have no items with a total item correlation below .30 as recommended (Stevens, 2009). In addition, only by removing item 3 of the SP scale (“I tend to be concerned with sexual matters”) can a slight improvement of Cronbach's alpha be observed for this subscale both in Colombia and Spain. However, their level of fitness is good and we do not deem its removal appropriate. Another good indicator is the distribution of the items where the scores – as expected for the population – have been higher than the theoretical mean for SSE and lower for SD and SP. Furthermore, deviations are close to 1, thereby indicating adequate variability (Carretero-Dios & Pérez, 2007).

An important aspect of this study has been the confirmation of the configural dimensionality of the scale. So far – to the best of our knowledge – only Wiedemann and Allgeier (1993) had tried to confirm its dimensionality, yet with little success. This study has reached a minimum level of invariance (configural invariance). While it is true that configural invariance would not allow comparisons between the two countries without bias, it does allow us to state that the scale is three-dimensional both in Colombia and Spain. In fact, this basic fit of this dimensional structure is excellent. The factor loadings are remarkably higher than .30 in both versions (except for item SP3, with the following values: .39 in the Colombian and .41 in the Spanish version), thereby indicating adequate construct validity again.

Concurrent validity is adequate to moderate and would find significant correlations with scales which evaluate other components of sexuality and self-esteem. Correlations between the SS components are consistent with other findings. The SSE subscale correlated negatively with SD as already observed (Snell et al., 2001; Snell & Papini, 1989; Wiedemann & Allgeier, 1993). These negative correlations suggest that sexual self-esteem and sexual depression are two opposite points of a continuum, so they are not really two different psychological dimensions of human sexuality (Snell & Papini, 1989). It is logical to think that people with high levels of sexual self-esteem are less likely to negatively evaluate aspects of their sex life, and to feel dissatisfied and unhappy with their sexuality (Snell et al., 2001; Sanchez-Fuentes et al, 2014.). As expected (Wiedemann & Allgeier, 1993), SSE correlates positively with the overall self-esteem thus demonstrating the usefulness of the test as a specific indicator of self-esteem, but not interchangeably with overall self-esteem (Rosenberg, Schooler, Schoenbach, & Rosenberg, 1995; Swenson, Houck, Barker, Zeanah, & Brown, 2012). Because people tend to differently evaluate different aspects of the self (Harter, 1982; Markus & Wurf, 1987), it is logical for a person to have a sense of the sexual self which differs from – though it contributes to – the overall sense of self (Zeanah & Schwarz, 1996). In turn, the negative correlation between overall self-esteem with SD is consistent with the idea that this may be an indicator of an instance of clinical depression in which low sexual interest and desire are a result thereof (Wiedemann & Allgeier, 1993).

Positive correlations of SSE and negative correlations of SD with MGH-SFQ and Initiation scales indicate good properties of the scale as a predictor of sexual functioning, as evidenced in other studies (Dove & Wiederman, 2000; Mezones-Holguin et al., 2011; Wentland et al., 2009) and sexual assertiveness (Ménard & Offman, 2009). In addition, this confirms the consistency of these constructs as opposed to each other. In other words, this reinforces the idea that people with high levels of SSE have higher levels of sexual assertiveness and sexual performance, and high levels of SD would indicate contrariwise. Correlations of greater magnitude have been observed in this sample of men and women between attitudes towards sexuality with SSE and SP than those observed only in women in previous studies (Kelley, 2012).

Since FI showed that the scales are not equivalent between the two cultures, no such comparisons have been made. However, statistically significant differences have been observed in SP between men and women, but not so between SSE and SD on the same line of Snell et al., (2001), Snell and Papini (1989) and Wiedemann and Allgeier (1993). There are differences in the SSE scale in the Colombian sample, between those who have a relationship and those who do not, which leads to positive correlations with sexual function and assertiveness. Similarly, the idea of evaluations that people make of their sexuality are associated with sexual satisfaction (Carrobles et al., 2011) and sexual wellbeing (Muise et al., 2010), which is achieved in relationships. No such differences are found in the Spanish sample, which could be explained by cultural differences in relation to marital status and the variables associated with religiousness.

In conclusion, the psychometric properties of the scales in the two versions, regarding reliability and validity, show that these scales can be useful tools to strengthen clinical and healthcare – related work in both countries. Their impact is demonstrated in relation to psychosocial functioning (Syme, Delaney, Schuster, Gosian, & Moye, 2013), with adolescent sexuality (Harden, 2014), with anxiety and sexual satisfaction (Brassard, Dupuy, Bergeron, & Shaver, 2015), with body and sexual satisfaction (Blodgett & Benson, 2013; La Rocque & Cioe, 2011; Van Den Brink et al., 2013). This makes these scales useful tools for evaluation and intervention.

Finally, some limitations encountered should be considered in future studies, as follows; the type of incidental sampling does not allow for generalization of data to the population of the two countries. The mechanism used for data collection is limited as there is no easy access to technological resources by certain sectors of the population; this implies an exclusion of data that could be significant and impactful. It is suggested to review item 3 of the SD scale, so as to more appropriately adjust to the theoretical parameters of the scale. Though its effect had no implications in this study, its appropriateness may improve the properties of the scale. One aspect to be evaluated in future research is the relationship of SP and SD with other components of human sexuality such as sexual abuse and victimization, physical attractiveness and sexual wellbeing, among others.

**References**

AERA, APA y NCME. (2014). *Standards for educational and psychological tests.* Washington DC: American Educational Research Association.

Aiken, L. R. (1980). Content validity and reliability of single items or questionnaires. *Educational and Psychological Measurement*, *40*, 955–959. doi: 10.1177/001316448004000419

Aiken, L. R. (1985). Three coefficients for analyzing the reliability and validity of ratings. *Educational and Psychological Measurement, 45*, 131-142. doi: 10.1177/0013164485451012

Andersen, B. L. (1999). Surviving Cancer: The importance of sexual self-concept. *Medical and Pediatric Oncology 33*, 15–23. doi: 10.1002/(SICI)1096-911X(199907)33:1<15::AID-MPO4>3.0.CO;2-L

Bentler, P. M. (1990). Comparative fit indexes in structural models. *Psychological Bulletin, 107,* 238-246. doi: 10.1037/0033-2909.107.2.238

Blodgett, E. H., & Benson, K. E. (2013) Differences in emerging-adult women's body image and sexuality outcomes according to BMI and dating status. *International Journal of Sexual Health, 25,* 225-239. doi: 10.1080/19317611.2013.801932

Brassard, A., Dupuy, E., Bergeron, S., & Shaver, P. (2015). Attachment insecurities and women’s sexual function and satisfaction: The mediating roles of sexual self-esteem, sexual anxiety, and sexual assertiveness. *Journal of Sex Research*, *52*, 110–119. doi: 10.1080/00224499.2013.838744

Calogero, R. M., & Thompson, J. K. (2009a) Potential implications of the objectification of women’s bodies for women’s sexual satisfaction. *Body Image, 6,* 145-148. doi:10.1016/j.bodyim.2009.01.001

Calogero, R. M., & Thompson, J. K. (2009b). Sexual self-esteem in American and British college women: relations with self-objectification and eating problems. *Sex Roles, 60*, 160-173. doi: 10.1007/s11199-008-9517-0

Carretero-Dios, H., & Pérez, C. (2007). Standards for the development and review of instrumental studies: Considerations about test selection in psychological research. *International Journal of Clinical and Health Psychology, 7,* 863-882.

Carrobles, J. A., Gámez-Guadix, M., & Almendros, C. (2011). Funcionamiento sexual, satisfacción sexual y bienestar psicológico y subjetivo en una muestra de mujeres españolas. *Anales de psicología*, *27*(1), 27-34.

# Cheung, G. W., & Rensvold, R. B. (2002). Evaluating goodness-of-fit indexes for testing measurement invariance. *Structural Equation Modeling*, *9,* 233-255. doi: 10.1207/S15328007SEM0902_5

Cleary, V., Hegarty, J., & McCarthy, G. (2011). Sexuality in irish women with gynecologic cancer. *Oncology Nursing Forum, 38,* E87-E96. doi: 10.1188/11.ONF.

Declaración de Helsinki de la Asociación médica mundial. (1964). Principios éticos para las investigaciones médicas en seres humanos.

Dove, N. L., & Wiederman, M. W. (2000). Cognitive distraction and women's sexual functioning. *Journal of Sex & Marital Therapy, 26*, 67-78. doi: 10.1080/009262300278650

Elosua, P., Mujika, J., Almeida, L. S., & Hermosilla, D. (2014). Procedimientos analítico-racionales en la adaptación de test. Adaptación al español de la batería de pruebas de razonamiento. *Revista Latinoamericana de Psicologia, 46*, 117- 126. doi:10.1016/S0120-0534(14)70015-9

Finkelhor, D. (1981). The sexual abuse of boys. *Victimology, 6,* 76–84

Fisher, W. A., White, L. A., Byrne, D., & Kelley, K. (1988): Erotophobia‐erotophilia as a dimension of personality. *The* *Journal of Sex Research*, *25*, 123-151. doi: 10.1080/00224498809551448

Gaynor, P., & Underwood, J. (1995). Conceptualizing and measuring sexual self-esteem. In: P. Shrout & S. Fiske (Eds.) *Personality Research, Methods and Theory*: A Festschrift Honoring Donald W. Fiske. Hillsdale, NJ: Erlbaum.

Giordano, F. G., & Rush, C. L. (2010). Self-esteem and sexuality: An exploration of differentiation and attachment. In M. H. Guindon (Ed.), *Self-esteem across the lifespan: Issues and interventions* (pp. 205–218). New York, NY: Routledge.

Gómez-Lugo, M., Espada, J., Morales, A., Marchal-Bertrand, L., Soler, F. y Vallejo-Medina, P. (2015). Adaptación, validación, fiabilidad y equivalencia factorial de la escala de autoestima de Rosenberg en población colombiana. Paper under revisión.

Harden, P. (2014). A Sex-Positive Framework for Research on Adolescent Sexuality. *Perspectives on Psychological Science, 9*, 455-469. doi: 10.1177/1745691614535934

Harter, S. (1982). The perceived competence scale for children. *Child Development, 53*, 87-97. doi: 10.2307/1129640

Heinrichs, K. D., MacKnee, C., Auton-Cuff, F., & Domene, J. F. (2009). Factors affecting sexual self-esteem among young adult women in long-term heterosexual relationships. *Canadian Journal of Human Sexuality, 18*(4), 183-199.

Hu, L., & Bentler, P. M. (1999). Cutoff criteria for fit indexes in covariance structure analysis: Conventional criteria versus new alternatives. *Structural Equation Modeling, 6,* 1-55. doi: 10.1080/10705519909540118

Informe Belmont. (1978). Principios éticos y pautas para la protección de los seres humanos en la investigación. Departamento de Salud, Educación y Bienestar de los Estados Unidos.

James, R. (2011). Correlates of sexual self-esteem in a sample of substance-abusing women. *Journal of Psychoactive Drugs, 43*, 220-228. doi: 10.1080/02791072.2011.605700

Kelley, E. (2012). *An examination of the roles of cognitive-affective sexual appraisals and coping strategies in the relationship between sexual victimization and sexual functioning*. Master’s dissertation, Ohio University.

Labbate, L. A., & Lare, S. B. (2001). Sexual dysfunction in male psychiatric outpatients: validity of the Massachusetts General Hospital Sexual Functioning Questionnaire. *Psychother Psychosom, 70*, 221-225.

La Rocque, C. L., & Cioe, J. (2011). An evaluation of the relationship between body image and sexual avoidance. *Journal of Sex Research, 48*, 397-408. doi: 10.1080/00224499.2010.499522

Lemieux, S. R., & Byers, E. S. (2008). The sexual well-being of women who have experienced child sexual abuse. *Psychology of Women Quarterly, 32*, 126–144. doi: 10.1111/j.1471-6402.2008.00418.x

McCabe, M., & Taleporos, G. (2003). Sexual esteem, sexual satisfaction, and sexual behavior among people with physical disability. *Archives of Sexual Behavior, 2003, 32*, 359-369.

Markus, H., & Wurf, E. (1987). The dynamic self-concept: A social-psychological perspective. *Annual Review of Psychology, 38*, 299-337. doi: 10.1146/annurev.ps.38.020187.001503

Marchal-Bertrand, L., Espada, J. P., Morales, A., Gómez-Lugo, M., Soler, F., & Vallejo-Medina, P. (2015). Adaptación, validación, fiabilidad del Massachussets General Hospital-Sexual Functioning Questionnaire en una muestra colombiana y equivalencia factorial con la versión española. (Paper under review)

Martín-Albo, J., Núñez, J., Navarro, J., & Grijalbo, F. (2007). The Rosenberg self-esteem: Translation and validation in university students. *The Spanish Journal of Psychology, 10*, 458-467. doi: http://dx.doi.org/10.1017/S1138741600006727

Mayers, K. S., Heller, D. K., & Heller, J. A. (2003) Damaged sexual self-esteem: A kind of disability. *Sexuality and Disability 21*, 269-282. doi: 10.1023/B:SEDI.0000010069.08844.04

Ménard, A., & Offman, A. (2009). The interrelationships between sexual self-esteem, sexual assertiveness and sexual satisfaction. *The Canadian Journal of Human Sexuality, 18*, 35-45.

Merino Soto, C., & Livia Segovia, J. (2009). Intervalos de confianza asimétricos para el índice la validez de contenido: Un programa Visual Basic para la V de Aiken. *Anales de Psicología, 25*, 169-171.

Mezones-Holguin, E., Córdova-Marcelo, W., Lau-Chu-Fon, F., Aguilar-Silva, C., Morales-Cabrera, J., Bolaños-Díaz, R., Pérez-López, F., & Chedraui, P. (2011). Association between sexual function and depression in sexually active, mid-aged, Peruvian women. *Climateric, 14*, 654-660. doi: 10.3109/13697137.2011.575480.

Montero, I., & León, O. G. (2007). A guide for naming research studies in Psychology. *International Journal of Clinical and Health Psychology, 7*, 847-862.

Morokoff, P. J., Quina, K., Harlow, L. L., Whitmire, L., Grimley, D. M., Gibson, P. R., & Burkholder, G. J. (1997). Sexual assertiveness scale (SAS) for women: Development and validation. *Journal of Personality and Social Psychology, 73*, 790-804. doi: 10.1037/0022-3514.73.4.790

Muise, A., Preyde, M., Maitland, S. B., & Milhausen, R. R. (2010). Sexual identity and sexual well-being in female heterosexual university students. *Archives of Sexual Behavior, 39,* 915-925. doi: 10.1007/s10508-009-9492-8

Muñiz, J., Elosua. P., & Hambleton, R. K. (2013). Directrices para la traducción y adaptación de los tests: Segunda edición. *Psicothema, 25,* 151-157.doi: 10.7334/psicothema2013.24

Nunnally, J. C. & Bernstein, I. J. (1995). *Teoría psicométrica*. Madrid: McGraw-Hill.

Osterlind, S. J. (1989). *Constructing Test Items*. Londres: Kluwer Academic Publishers.

Reed, R. S. (1988). *Romantic and sexual self-esteem scale*. Unpublished manuscript, University of Nebraska-Lincoln.

República de Colombia. (1993). Resolución 8430 de 1993 acerca de la Investigación en Salud. Bogotá.

República de Colombia. (2006). Ley 1090 que reglamenta el ejercicio de la profesión de Psicología, dicta el Código Deontológico y Bioético y otras disposiciones. Bogotá.

Riggio, H., Galaz, B., Garcia, A., & Matthies, B. (2014). Contraceptive attitudes and sexual self-esteem among young adults: communication and quality of relationships with mothers. *International Journal of Sexual Health*, *26*, 268–281. doi: 10.1080/19317611.2014.885924

Robinson, B. E., Bockting, W. O., Rosser, B. R., Miner, M., & Coleman, E. (2002). The sexual health model: Application of a sexological approach to HIV prevention. *Health Education Research, 17*, 43–57. doi: 10.1093/her/17.1.43

Rosenberg, M. (1965). *Society and the Adolescent Self-image*. Princeton University Press: Princeton, NJ.

Rosenberg, M. (1989). *Society and the adolescent self-image*. (Rev.ed.). Middeltown, CT: Wesleyan University Press.

Rosenberg, M., Schooler, C., Schoenbach, C., & Rosenberg, F. (1995). Global self-esteem and specific self-esteem: Different concepts, different outcomes. *American Sociological Review, 60*, 141-156. doi: 10.2307/2096350

Rosenthal, D., Moore, S., & Flynn, I. (1991). Adolescent self-efficacy, self-esteem and sexual risk-taking. *Journal of Community & Applied Social Psychology, 1,* 77–88. doi: 10.1002/casp.2450010203

Sánchez-Fuentes, M., Santos-Iglesias, P., & Sierra, J. C. (2014). A systematic review of sexual satisfaction. *International Journal of Clinical and Health Psychology, 14*, 67-75.

Santos Iglesias, P., Sierra, J. C., García, M., Martínez, A., Sánchez, A., & Tapia, M. I. (2009). Índice de satisfacción sexual (ISS): Un estudio sobre su fiabilidad y validez. *International Journal of Psychology and Psychological Therapy, 9,* 259-273.

Santos-Iglesias, P., y Sierra, J. C (2010). El papel de la asertividad sexual en la sexualidad humana: una revisión sistemática. *International Journal of Clinical and Health Psychology, 10,* 553-577.

Seal, A., Minichiello, V., & Omodei, M. (1997). Young women´s sexual risk taking behaviour: re-visiting the influences of sexual self-efficacy and sexual self-esteem. *International Journal of STD & AIDS, 8*, 159-165. doi: 10.1258/0956462971919822

Schick, V. R., Calabrese, S. K., Rima, B. N. & Zucker, A. N. (2010). Genital appearance dissatisfaction: Implications for women's genital image self-consciousness, sexual esteem, sexual satisfaction, and sexual risk. *Psychology of Women Quarterly*, *34*, 394-404. **doi: 1**0.1111/j.1471-6402.2010.01584.x

**Sierra, J. C., Vallejo-Medina, P., Santos-Iglesias, P., & Lameiras, M. (2012).** Validation of Massachusetts General Hospital-Sexual Functioning Questionnaire (MGH-SFQ) in a Spanish population. *Atención Primaria, 44*, 516-524. doi: 10.1016/j.aprim.2012.02.004

**Sierra, J. C., Vallejo-Medina, P., & Santos-Iglesias, P. (2011). Propiedades psicométricas de la versión española de la Sexual Assertiveness Scale (SAS). *Anales de Psicología, 27*, 17-26.**

Sierra, J. C, Santos-Iglesias, P., & Vallejo-Medina, P. (2012). Evaluación de la equivalencia factorial y métrica de la Sexual Assertiveness Scale (SAS) por sexo. *Psicothema*, *24*, 316-322.

Snell, W. E., Jr., Fisher, T. D., & Schuh, T.  (2001). Chapter 3:  Reliability and validity of the Sexuality Scale: A measure of sexual-esteem, sexual-depression, and sexual-preoccupation.  In W. E. Snell, Jr. (Ed.), *New directions in the psychology of human sexuality:  Research and theory*. Cape Girardeau, MO: Snell Publications. WEB: http://cstl-cla.semo.edu/snell/books/sexuality/sexuality.htm.

**Snell, W. E., & Papini, D. (1989). The Sexuality Scale (SS): An instrument to measure sexual- esteem, sexual-depression, and sexual-preoccupation. *The Journal of Sex Research, 26*, 256-263.** doi: 10.1080/00224498909551510

Stevens, J.P. (2009). *Applied multivariate statistics for the social science* (5 Ed.). Mahwah, New Jersey: Lawrence Erlbaum Associates.

Struckman-Johnson, C., & Struckman-Johnson, D. (1994). Men pressured and forced into sexual experience. *Archives of Sexual Behavior, 23*, 93-114. doi: 0.1007/BF01541620

Swenson, R. R., Houck, C. D., Barker, D., Zeanah, P. D., & Brown, L. K. (2012). Prospective analysis of the transition to sexual experience and changes in sexual self-esteem among adolescents attending therapeutic schools. *Journal of Adolescence*, *35*, 77-85. doi: 10.1016/j.adolescence.2011.06.002

Syme, M., Delaney, E., Schuster, J., Gosian, J., & Moye, J. (2013) Sexual Self-Esteem and psychosocial functioning in military veterans after cancer. *Journal of Psychosocial Oncology, 31*, 1-12. doi: 10.1080/07347332.2012.741096

Vallejo-Medina, P., Granados, R., & Sierra, J. C. (2014). Propuesta y validación de una versión breve de la Sexual Opinion Survey en población española. *Revista Internacional de Andrología, 12*, 47-54. doi: 10.1016/j.androl.2013.04.004

Vallejo-Medina, P., Marchal-Bertrand, L., Gómez-Lugo, M., Espada, J.P., Sierra, J., Soler, F. y Morales, A. (2015). Adaptation and validation of the brief sexual opinion survey (SOS) in a Colombian sample and factorial equivalence with the Spanish version. Paper under revision.

Vallejo-Medina, P., & Sierra, J. C. (2015). Adaptation and validation of the Sexual Assertiveness Scale (SAS) in a sample of male drug users. *The Spanish Journal of Psychology, 18*, E21. doi:10.1017/sjp.2015.25

Van Bruggen, L., Runtz, M., & Kadlec, H. (2006). Sexual revictimization: The role of sexual self-esteem and dysfunctional sexual behaviors. *Child Maltreatment, 11,* 131-145. doi: 10.1177/1077559505285780

Van Den Brink, F., Smeets, M. A., Hessen, D. J., Talens, J. G., & Woertman, L. (2013). Body satisfaction and sexual health in Dutch female university students. *Journal of Sex Research, 50, 8*, 786-794. doi: 10.1080/00224499.2012.684250

Wentland, J. J., Herold, E. S., Desmarais, S., & Milhausen, R. R. (2009). Differentiating highly sexual women from less sexual women. *Canadian Journal of Human Sexuality, 18*, 169-182.

Wiederman, M. W., & Allgeier, E. R. (1993). The measurement of sexual-esteem: Investigation of Snell and Papini's (1989) Sexuality Scale. *Journal of Research in Personality*, *27*, 88-102. doi: 10.1006/jrpe.1993.1006

Wiederman, M. W. & Hurst, S. R. (2010) Body size, physical attractiveness, and body image among young adult women: Relationships to sexual experience and sexual esteem. *Journal of Sexual Research, 35,* 272-281. doi: 10.1080/00224499809551943

Yuan, K. H., & Bentler, P. M. (2000). Three likelihood-based methods for mean and covariance structure analysis with non-normal missing data. In M. E. Sobel & M. P. Becker (Eds.), *Sociological methodology 2000* (pp. 165–200). Washington, DC: American Sociological Association.

Zeanah, P. D., & Schwarz, J. C. (1996). Reliability and Validity of the Sexual Self-Esteem Inventory for Women. *Assessment, 3*, 1-15. doi: 10.1177/107319119600300101

Table 1.

*Characteristics of the Participants’ Sample in Colombia and Spain.*

| Variable | | Colombia | Spain | Hypothesis contrast | |
| --- | --- | --- | --- | --- | --- |
| Sex |  |  |  |  |  |
|  | Female | 397 | 313 | *χ^2^(1) = .15; p = .69* | |
|  | Male | 242 | 200 |  |  |
| Age ranges | |  |  |  |  |
| 1 | 18 - 30 | 309 | 268 | *χ^2^(2) = 6.51; p = .039; η =* 0.003 | |
| 2 | 31 - 44 | 235 | 154 |  |  |
| 3 | Older than 45 | 102 | 99 |  |  |
| Years of schooling | | 16.70 (2.85) | 15.77 (4.07) | *t(1154) = 4.53; p ˂ .01; d = .26* | |
|  | |  |  |  |  |
|  | 1 | 553 | 432 | *χ^2^(7) = 9.02; p = .25* | |
|  | 2 | 41 | 38 |  |  |
|  | 3 | 10 | 5 |  |  |
|  | 4 | 7 | 6 |  |  |
|  | 5 | 0 | 4 |  |  |
|  | 6 | 9 | 12 |  |  |
|  | 7 | 17 | 20 |  |  |
|  | 8 | 4 | 3 |  |  |
| Relationship | |  |  |  |  |
|  | Yes | 454 | 371 | *χ^2^(1) = .06; p = .81* | |
|  | No | 188 | 149 |  |  |
| Marital status | |  |  |  |  |
|  | Single | 359 | 313 | *χ^2^(3) = 22.16; p < .01; η =* 0.094 | |
|  | Married | 151 | 144 |  |  |
|  | Common-law marriage | 87 | 30 |  |  |
|  | Divorced | 44 | 25 |  |  |
| Religion | |  |  |  |  |
|  | Christian | 333 | 245 | *χ^2^(6) = 101.61; p < .01; η =* 0.003 | |
|  | Jewish | 2 | 2 |  |  |
|  | Hindu | 1 | 1 |  |  |
|  | Buddhist | 5 | 0 |  |  |
|  | Catholic | 85 | 0 |  |  |
|  | None | 206 | 268 |  |  |
|  | Other | 5 | 2 |  |  |
| Religiousness | |  |  |  |  |
|  | Daily | 5 | 1 | *χ^2^(4) = 189.41; p < .01; η =* 0.389 | |
|  | Once a week | 104 | 13 |  |  |
|  | Rarely a month | 149 | 23 |  |  |
|  | Rarely a year | 214 | 194 |  |  |
|  | Never | 165 | 287 |  |  |

*Note*: Sexual orientation was evaluated on a continuum from 1 (exclusively heterosexual) to 7 (exclusively homosexual); χ 2 = Chi square

 Table 2.

Psychometric Properties of the Sexuality Scale

| Subscale | Item | Colombia | | | | | | |  | Spain | | | | | | |
| --- | --- | --- | --- | --- | --- | --- | --- | --- | --- | --- | --- | --- | --- | --- | --- | --- |
|  |  | *M* | *SD* | *r*_it_^c^ | *α - i* | *α* | *Total M* | *Total SD* |  | *M* | *SD* | *r*_it_^c^ | *α - i* | *α* | *Total M* | *Total SD* |
| SSE | SSE1 | 4.42 | 0.90 | .59 | .81 | .83 | 21.69 | 3.49 |  | 4.10 | 0.93 | .71 | .84 | .87 | 20.31 | 3.95 |
|  | SSE2 | 4.07 | 0.89 | .69 | .78 |  |  |  |  | 3.96 | 0.91 | .76 | .83 |  |  |  |
|  | SSE3 | 4.32 | 0.86 | .77 | .76 |  |  |  |  | 3.87 | 0.97 | .81 | .82 |  |  |  |
|  | SSE4 | 4.53 | 0.88 | .56 | .81 |  |  |  |  | 4.39 | 0.95 | .56 | .88 |  |  |  |
|  | SSE5 | 4.36 | 0.99 | .54 | .82 |  |  |  |  | 4.00 | 1.10 | .67 | .86 |  |  |  |
| SD | SD1 | 1.75 | 1.19 | .66 | .84 | .87 | 8.29 | 4.45 |  | 1.93 | 1.22 | .68 | .83 | .86 | 9.61 | 4.91 |
|  | SD2 | 1.47 | 0.90 | .57 | .86 |  |  |  |  | 1.53 | 0.96 | .45 | .88 |  |  |  |
|  | SD3 | 1.77 | 1.22 | .71 | .83 |  |  |  |  | 2.18 | 1.38 | .75 | .82 |  |  |  |
|  | SD4 | 1.61 | 1.12 | .78 | .81 |  |  |  |  | 1.85 | 1.29 | .81 | .80 |  |  |  |
|  | SD5 | 1.69 | 1.05 | .73 | .83 |  |  |  |  | 2.13 | 1.23 | .73 | .82 |  |  |  |
| SP | SP1 | 2.79 | 1.25 | .69 | .80 | .84 | 11.22 | 4.89 |  | 2.43 | 1.24 | .73 | .81 | .85 | 10.28 | 4.76 |
|  | SP2 | 1.90 | 1.17 | .69 | .80 |  |  |  |  | 1.74 | 1.13 | .74 | .81 |  |  |  |
|  | SP3 | 1.99 | 1.19 | .36 | .88 |  |  |  |  | 1.96 | 1.20 | .40 | .89 |  |  |  |
|  | SP4 | 2.26 | 1.29 | .78 | .78 |  |  |  |  | 2.00 | 1.20 | .74 | .80 |  |  |  |
|  | SP5 | 2.28 | 1.32 | .77 | .78 |  |  |  |  | 2.14 | 1.22 | .75 | .80 |  |  |  |

*Note:* SSE = Sexual Self-Esteem; SD: Sexual Depression; SP: Sexual Preoccupation; *M* = Mean; *SD =* S; *r*_it_^c^ = Corrected item-total correlations; α-i = Cronbach alpha if the item is deleted; α: = Cronbach alpha.

Table 3

*Factor loadings of the Sexuality Scale Items for Colombia and Spain versions*

| Items | Factor | Colombia | | |  | Spain | | |
| --- | --- | --- | --- | --- | --- | --- | --- | --- |
|  |  | *λ* | *Error* | *R^2^* |  | *λ* | *Error* | *R^2^* |
| SSE1 | SSE | .65 | .76 | .42 |  | .76 | .65 | .57 |
| SSE2 |  | .81 | .58 | .66 |  | .85 | .53 | .72 |
| SSE3 |  | .88 | .49 | .77 |  | .91 | .42 | .83 |
| SSE4 |  | .61 | .79 | .37 |  | .59 | .81 | .34 |
| SSE5 |  | .57 | .82 | .32 |  | .70 | .72 | .49 |
| SD1 | SD | .71 | .70 | .50 |  | .74 | .67 | .55 |
| SD2 |  | .61 | .79 | .38 |  | .49 | .87 | .24 |
| SD3 |  | .77 | .64 | .60 |  | .84 | .54 | .71 |
| SD4 |  | .85 | .53 | .72 |  | .89 | .46 | .79 |
| SD5 |  | .80 | .60 | .64 |  | .79 | .62 | .62 |
| SP1 | SP | .76 | .65 | .58 |  | .81 | .59 | .65 |
| SP2 |  | .76 | .65 | .58 |  | .83 | .57 | .68 |
| SP3 |  | .39 | .92 | .15 |  | .41 | .91 | .17 |
| SP4 |  | .85 | .53 | .72 |  | .83 | .56 | .69 |
| SP5 |  | .88 | .48 | .77 |  | .81 | .58 | .66 |

*Note:* SSE = Sexual Self-Esteem; SD: Sexual Depression; SP: Sexual Preoccupation; λ = Factorial loading; *R^2^ =* variance of the item explained by factor.

Table 4

*Correlations of the two Versions of the Sexuality Scale with other Scales*

| Colombia | SSE | SD | SP | In | Re | STD_P | SOS | Self Gral | MGHSFQ |
| --- | --- | --- | --- | --- | --- | --- | --- | --- | --- |
| Spain |  |  |  |  |  |  |  |  |  |
| SSE | 1.00 | **-.51^**^** | **.06** | **.30^**^** | **-0,07** | **-.12^**^** | **.11^**^** | **.34^**^** | **.33^**^** |
| SD | **-.55^**^** | 1.00 | **.10^*^** | **-.30^**^** | **.08** | **.03** | **.02** | **-.39^**^** | **-.49^**^** |
| SP | **.05** | **.17^**^** | 1.00 | **.10^*^** | **-.16^**^** | **-.09^*^** | **.27^**^** | **-.11^**^** | **.19^**^** |
| In | **.35^**^** | **-.39^**^** | **-.03** | 1.00 | .11^**^ | .04 | .30^**^ | .22^**^ | .23^**^ |
| Re | **-.01** | **-.07** | **-.22^**^** | .23^**^ | 1.00 | .22^**^ | -.02 | -.05 | -.22^**^ |
| STD_P | **-.07** | **.01** | **-.07** | .10^*^ | .20^**^ | 1.00 | -.01 | -.04 | .01 |
| SOS | **.10^*^** | **-.09** | **.09^*^** | .20^**^ | .05 | .02 | 1.00 | -.01 | .05 |
| Self Gral | **.41^**^** | **-.41^**^** | **-.09** | .22^**^ | .00 | -.07 | .06 | 1.00 | .32^**^ |
| MGHSFQ | **.28^**^** | **-.40^**^** | **.20^**^** | .14^**^ | -.23^**^ | -.04 | .02 | .29^**^ | 1.00 |

*Note:* SSE: Sexual Self-Esteem; SD: Sexual Depression; SP: Sexual Preoccupation; In: Sexual Assertiveness Initiation; Re: Sexual Assertiveness Refusal; STD-P = Sexual Assertiveness to negotiate the use of condom (to prevent unwanted pregnancy and sexually transmitted diseases); SOS: attitudes towards sexuality (erotophobia-erotophilia); Self Gral: General Self-Esteem; MGHSFQ: Sexual Functioning Questionnaire (Massachusetts General Hospital**);** * = *p* < .05; ** = *p* < .01. Correlations with SSE, SD and SP are marked in bold.

**Appendix**

*Version Validated in Colombia of the Sexuality Scale (SS;* Snell & Papini 1989) *Abbreviated Scale* (Wiedeman & Allgeier, 1993).

1 = En total desacuerdo; 2 = Medianamente en desacuerdo; 3 = Ni de acuerdo, ni en desacuerdo; 4 = Medianamente de acuerdo; 5 = Totalmente de acuerdo

| 1. Soy una buena pareja sexual | 1 | 2 | 3 | 4 | 5 |
| --- | --- | --- | --- | --- | --- |
| 1. Yo calificaría mi habilidad sexual como muy alta | 1 | 2 | 3 | 4 | 5 |
| 1. Pienso que soy una muy buena pareja a nivel sexual | 1 | 2 | 3 | 4 | 5 |
| 1. Tengo confianza en mí mismo como pareja sexual | 1 | 2 | 3 | 4 | 5 |
| 1. Yo me calificaría en un nivel bajo como pareja sexual | 1 | 2 | 3 | 4 | 5 |
| 1. Me siento cómodo con mi sexualidad | 1 | 2 | 3 | 4 | 5 |
| 1. Me siento deprimido por los aspectos sexuales de mi vida | 1 | 2 | 3 | 4 | 5 |
| 1. Me siento triste con mi vida sexual | 1 | 2 | 3 | 4 | 5 |
| 1. Me siento decepcionado por la calidad de mi vida sexual | 1 | 2 | 3 | 4 | 5 |
| 1. Me siento feliz con mi vida sexual | 1 | 2 | 3 | 4 | 5 |
| 1. Pienso en sexo todo el tiempo | 1 | 2 | 3 | 4 | 5 |
| 1. Tiendo a estar preocupado con temas sexuales | 1 | 2 | 3 | 4 | 5 |
| 1. Pienso en sexo más que en cualquier otra cosa | 1 | 2 | 3 | 4 | 5 |
| 1. Estoy constantemente pensando en tener sexo | 1 | 2 | 3 | 4 | 5 |
| 1. Pienso en sexo una gran parte del tiempo | 1 | 2 | 3 | 4 | 5 |

*Version Validated in Spain of the Sexuality Scale (SS;* Snell & Papini 1989) *Abbreviated Scale* (Wiedeman & Allgeier, 1993).

1 = En total desacuerdo; 2 = Medianamente en desacuerdo; 3 = Ni de acuerdo, ni en desacuerdo; 4 = Medianamente de acuerdo; 5 = Totalmente de acuerdo

| 1. Soy una buena pareja sexual | 1 | 2 | 3 | 4 | 5 |
| --- | --- | --- | --- | --- | --- |
| 1. Considero que mis habilidades sexuales son muy buenas | 1 | 2 | 3 | 4 | 5 |
| 1. Me considero una pareja sexual muy buena | 1 | 2 | 3 | 4 | 5 |
| 1. Me percibo como una mala pareja sexual | 1 | 2 | 3 | 4 | 5 |
| 1. Tengo confianza en mí mismo/a como pareja sexual | 1 | 2 | 3 | 4 | 5 |
| 1. Me siento cómodo con mi sexualidad | 1 | 2 | 3 | 4 | 5 |
| 1. Me deprimen los aspectos sexuales de mi vida | 1 | 2 | 3 | 4 | 5 |
| 1. Me entristece mi vida sexual | 1 | 2 | 3 | 4 | 5 |
| 1. Me decepciona la calidad de mi vida sexual | 1 | 2 | 3 | 4 | 5 |
| 1. Estoy feliz con mi vida sexual | 1 | 2 | 3 | 4 | 5 |
| 1. Pienso en sexo todo el tiempo | 1 | 2 | 3 | 4 | 5 |
| 1. Tiendo a estar preocupado por el sexo | 1 | 2 | 3 | 4 | 5 |
| 1. Pienso en sexo más que en ninguna otra cosa | 1 | 2 | 3 | 4 | 5 |
| 1. Constantemente pienso en practicar sexo | 1 | 2 | 3 | 4 | 5 |
| 1. Pienso sobre temas sexuales una gran parte del tiempo | 1 | 2 | 3 | 4 | 5 |
